# The ADIPS Pilot National Diabetes in Pregnancy Benchmarking Programme

**DOI:** 10.3390/ijerph18094899

**Published:** 2021-05-04

**Authors:** Jincy Immanuel, Jeff Flack, Vincent W Wong, Lili Yuen, Carl Eagleton, Dorothy Graham, Janet Lagstrom, Louise Wolmarans, Michele Martin, Ngai Wah Cheung, Suja Padmanabhan, Victoria Rudland, Glynis Ross, Robert G Moses, Louise Maple-Brown, Ian Fulcher, Julie Chemmanam, Christopher J Nolan, Jeremy J N Oats, Arianne Sweeting, David Simmons

**Affiliations:** 1 School of Medicine, Western Sydney University, 2560 Sydney, Australia; j.varghese@westernsydney.edu.au (J.I.); Jeff.Flack@health.nsw.gov.au (J.F.); 18823501@student.westernsydney.edu.au (L.Y.); 2 Department of Diabetes and Endocrinology, Bankstown-Lidcombe Hospital, 2200 Sydney, Australia; 3 South Western Sydney Clinical School, University of New South Wales, 2170 Liverpool, Australia; vincent.wong1@health.nsw.gov.au; 4 Diabetes and Endocrinology Service, Liverpool Hospital, 2170 Sydney, Australia; 5 Department of Endocrinology, Auckland City Hospital, 1023 Auckland, New Zealand; CarlE@adhb.govt.nz; 6 Obstetrics and Gynaecology, King Edward Memorial Hospital, University of Western Australia, 6008 Subiaco, Australia; dorothy.graham@health.wa.gov.au; 7 Nathalia Cobram Numurkah Health, 3636 Victoria, Australia; Janet.Lagstrom@ncnhealth.org.au; 8 Diabetes Service, Waikato Hospital, 3204 Hamilton, New Zealand; Louise.Wolmarans@waikatodhb.health.nz; 9 Diabetes Service, Illawarra Shoalhaven Local Health District, 2500 Wollongong, Australia; Michele.Martin@health.nsw.gov.au (M.M.); bmoses@bigpond.net.au (R.G.M.); 10 Department of Diabetes and Endocrinology, Westmead Hospital, 2145 Sydney, Australia; wah.cheung@sydney.edu.au (N.W.C.); suja.padmanabhan@sydney.edu.au (S.P.); victoria.rudland@sydney.edu.au (V.R.); 11 Department of Diabetes and Endocrinology, Royal Prince Alfred Hospital, 2050 Sydney, Australia; gpross@bigpond.net.au (G.R.); arianne.sweeting@sydney.edu.au (A.S.); 12 Menzies School of Health Research, Charles Darwin University, 0810 Darwin, Australia; Louise.Maple-Brown@menzies.edu.au; 13 Department of Endocrinology, Royal Darwin Hospital, 0810 Darwin, Australia; 14 Department of Obstetrics and Gynaecology, Liverpool Hospital, 2170 Sydney, Australia; IAN.FULCHER@health.nsw.gov.au; 15 Endocrinology and Diabetes Centre, Women’s and Children’s Hospital, 5006 Adelaide, Australia; julie.chemmanam@sa.gov.au; 16 Department of Diabetes and Endocrinology, The Canberra Hospital, 2605 Garran, Australia; christopher.nolan@anu.edu.au; 17 Medical School, Australian National University, 2605 Canberra, Australia; 18 Melbourne School of Population and Global Health, University of Melbourne, 3053 Victoria, Australia; jjnoats@gmail.com; 19 Macarthur Diabetes Service, Campbelltown Hospital, 2560 Sydney, Australia

**Keywords:** gestational diabetes mellitus, healthcare benchmarking, audit, pregnancy, type 1 diabetes mellitus, type 2 diabetes mellitus, pregnancy outcomes, HbA1c

## Abstract

Background: To test the feasibility of benchmarking the care of women with pregnancies complicated by hyperglycaemia. Methods: A retrospective audit of volunteer diabetes services in Australia and New Zealand involving singleton pregnancies resulting in live births between 2014 and 2020. Ranges are shown and compared across services. Results: The audit included 10,144 pregnancies (gestational diabetes mellitus (GDM) = 8696; type 1 diabetes (T1D) = 435; type 2 diabetes (T2D) = 1013) from 11 diabetes services. Among women with GDM, diet alone was used in 39.4% (ranging among centres from 28.8–57.3%), metformin alone in 18.8% (0.4–43.7%), and metformin and insulin in 10.1% (1.5–23.4%); when compared between sites, all *p* < 0.001. Birth was by elective caesarean in 12.1% (3.6–23.7%) or emergency caesarean in 9.5% (3.5–21.2%) (all *p* < 0.001). Preterm births (<37 weeks) ranged from 3.7% to 9.4% (*p* < 0.05), large for gestational age 10.3–26.7% (*p* < 0.001), admission to special care nursery 16.7–25.0% (*p* < 0.001), and neonatal hypoglycaemia (<2.6 mmol/L) 6.0–27.0% (*p* < 0.001). Many women with T1D and T2D had limited pregnancy planning including first trimester hyperglycaemia (HbA1c > 6.5% (48 mmol/mol)), 78.4% and 54.6%, respectively (*p* < 0.001). Conclusion: Management of maternal hyperglycaemia and pregnancy outcomes varied significantly. The maintenance and extension of this benchmarking service provides opportunities to identify policy and clinical approaches to improve pregnancy outcomes among women with hyperglycaemia in pregnancy.

## 1. Introduction

Hyperglycaemia in pregnancy is a growing problem worldwide [1], posing significant risk to women and their pregnancies [2,3]. In Australia, more than one in 10 pregnancies are complicated by hyperglycaemia [4]. The risks of hyperglycaemia and related adverse pregnancy outcomes are greater among women of Indigenous background, and those born outside Australia [4,5]. Adverse events remain high in pregnancies complicated by diabetes [4,5,6,7], suggesting insufficient pre-pregnancy preparation and antenatal management. Profound urban–rural disparities [6] and variation in obstetric care across Australia [8] limit access to optimal care for many women. Similar risks and variation exist in New Zealand, with higher rates of gestational diabetes mellitus (GDM) and type 2 diabetes (T2D) in pregnancy among Māori and Pacific people [9].

Mapping variations in antenatal diabetes care is an important tool for improving the quality of care provided. A national audit and benchmarking program using standardised criteria was found to be useful for identifying aspects of pre-pregnancy and antenatal care needing improvement among women with diabetes [10], including the need for systemic change [11,12]. A national audit and benchmarking process is currently lacking in Australia for pregnancy care however is available outside of pregnancy [13]. In 1999, the Australasian Diabetes in Pregnancy Society (ADIPS) proposed a national diabetes in pregnancy clinical audit program to lead to the establishment of an internal audit and benchmarking process. Subsequently, a pilot audit conducted in 2003 showed the feasibility of a large-scale audit, but without funding, the approach was not sustainable [14]. The main objective of this current pilot was to develop a sustainable dataset for pregnancies complicated by hyperglycaemia, and to assist the development of a national clinical benchmarking and audit program. 

## 2. Materials and Methods

### 2.1. Participants

This multicentre pilot audit was conducted under the governance of ADIPS. All diabetes in pregnancy services across Australia and New Zealand, including those from tertiary, rural, indigenous, public, and private settings were eligible to participate in the pilot program. All members of ADIPS were invited to participate by the ADIPS secretariat via email, website, and forums. Other health facilities were open for participation upon approaching the Secretariat. Our target for the pilot was to include 10 centres from diverse settings. The benchmarking centre was located in a metropolitan city with a large multiethnic population and had expertise from prior benchmarking analyses outside of pregnancy. The benchmarking centre also volunteered to be one of the pilot sites. Pregnancies in women with GDM, type 1 diabetes (T1D), or T2D resulting in live births between years 2014 and 2020 were eligible for inclusion in the audit. Data from women with multiple pregnancies or pregnancies ending in miscarriage, induced abortion, or stillbirth were excluded from the audit because of variations in sources. 

### 2.2. Development of the Audit Tool

The audit tool was built upon the data set used previously [14], and the outcome variables were defined according to the International Association of the Diabetes and Pregnancy Study Groups’ consensus guidelines [15]. The audit tool was created in Excel and discussed at the 2019 ADIPS annual scientific meeting. The data items were further discussed using a Delphi process [16] and agreed to by the ADIPS Diabetes and Pregnancy Audit Working Group consisting of 17 specialists across Australia and New Zealand. The audit tool consisted of 55 items. Of those, 20 were core data items related to pregnancy planning and management to compare quality of pregnancy care against key recommendations in the ADIPS guidelines [17] (Table 1). 

The core data elements were complemented by demographic, treatment, and pregnancy outcome variables. Data items that were not specific to pregnancy were created based on nationally endorsed metadata standards accessible through a metadata online registry [18]. The data items are shown in Table 2. 

### 2.3. Data Collection

This pilot was undertaken in a “double blind” (site and participant) fashion. Data collection was coordinated by a trusted third party (TTP) process, where in this instance, a “trusted” agent acts as a channel for communication ensuring that participating sites remain anonymous to the benchmarking centre. Health services that volunteered to participate in the programme sent their existing locally approved data directly to the TTP after obtaining approval based on local policies. Data were deidentified before being sent to the benchmarking centre and were sent in a single Excel spreadsheet in standard format as an email attachment. Data were checked for completeness and formatting, and further queries were made to participating sites through the TTP for clarification as needed. All questioned data items that sites could not validate were deleted. All records containing births recorded as less than 28 weeks gestation were also deleted due to variations in data collection between sites. We accepted validated heights as low as 140 cm and as high as 185 cm and a validated weight maximum of 185 kg to avoid including data entry errors. The TTP created a unique identifier for each participating health service before disclosing the data for analysis to the benchmarking centre. Additionally, sites were re-coded at the central analysis site as an added security measure to preclude identification of any site from the analyses.

### 2.4. Feedback Reports

Participating sites were provided with a feedback report describing clinical care in their centre. The benchmarking report enabled them to compare their performance against other participating sites and identify local service needs. Sites were able to identify only their own data reports. 

### 2.5. Statistical Analysis

Comparisons were made between sites, but not between types of diabetes in pregnancy because of the selective nature of the audits. Outcome and other measures were reported based upon non-missing data. Sites having all data missing for a given variable were excluded from analyses relating to that variable. For categorical data, if the sites had reported only “Yes” values and the remaining cells were blank/missing, these were interpreted as “No”. Categorical variables were described using numbers and percentages and Pearson’s Chi-squared test, where appropriate, were used to compare groups. Continuous variables were described using mean ± standard deviation and compared using analysis of variance. Analyses were performed using IBM SPSS Version 24.0. Armonk, NY: IBM Corp. All tests are 2 tailed with *p* < 0.05 taken as significant. The ethics approval for this project was granted by the Western Sydney University Human Research Ethics Committee (HREC Approval Number: H13648).

## 3. Results

**Figure 1:**
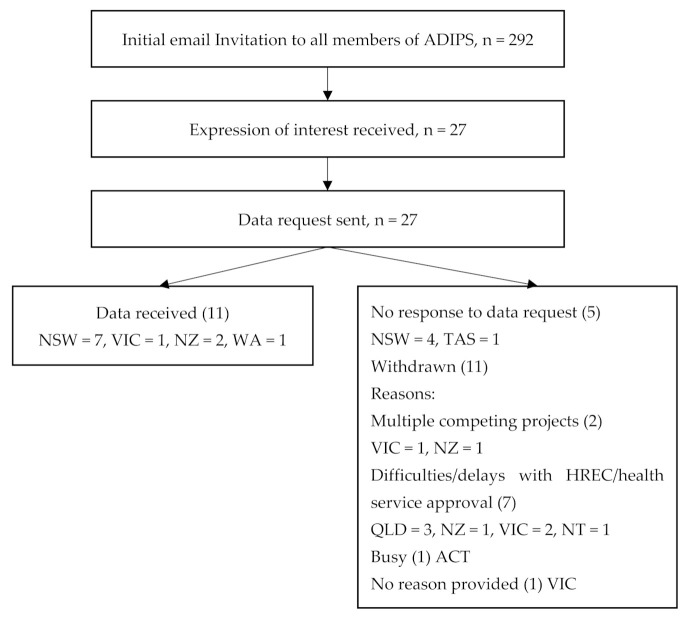
Flow chart of site selection process. ADIPS = Australasian Diabetes in Pregnancy Society; NSW = New South Wales, VIC = Victoria, NZ = New Zealand, WA = Western Australia; TAS = Tasmania, HREC = Human Research Ethics Committee; QLD = Queensland, NT = Northern Territory, ACT = Australian Capital Territory.

This pilot audit covered 10,144 patient records (GDM, *n* = 8696, T1D, *n* = 435, T2D, *n* = 1013) from 11 diabetes services (public clinics within tertiary urban hospitals with 2000–9000 births annually (*n* = 9), private urban clinic (*n* = 1), rural health service (*n* = 1)) after excluding multiple pregnancies (*n* = 103); pregnancies affected by new-onset diabetes after organ transplantation (*n* = 2) and maturity-onset diabetes of the young (*n* = 1); and pregnancies that ended in miscarriages, termination of pregnancy or perinatal death (*n* = 59) (Figure 1). The majority (7/11) of the participating sites were located in one Australian state (Figure 1). Eight services provided data for women with GDM, seven services reported data for T1D in pregnancy, and nine services reported data for T2D in pregnancy. Each site provided 1–5 years of data.

### 3.1. Maternal Characteristics

Table 3 shows the maternal characteristics of the study population. The mean age of the participants was 32.3 ± 5.5 years. The mean gravidity and parity across all sites were 2.9 ± 1.9 and 1.3 ± 1.4 respectively. The pre-pregnancy and booking weights were recorded for 64% and 61% of the reported pregnancies, respectively. The mean self-reported pre-pregnancy and booking weights were 74.7 ± 21.1 kg and 84.0 ± 23.5 kg, respectively. The mean pre-pregnancy body mass index for women with GDM, T1D, and T2D was 28.1 ± 7.0, 26.0 ± 5.4, and 31.5 ± 7.1 kg/m^2^, respectively. Within centres reporting these variables, 6.7% of the women documented having a history of hypertension and 9.1% reported smoking during pregnancy. Ethnicity was reported for 93% of women with significant variation in ethnic makeup across services. Most were European (33.1%), followed by South Asian (20.8%), East/South East Asian (13.8%), Māori/Pacific (12.5%), and Middle Eastern (8.1%). Aboriginal and Torres Strait Islander constituted 2.6% of women. 

### 3.2. Gestational Diabetes Mellitus

Table 4 shows the missing data, number of participating services, the proportion or mean with the benchmark measure and the range across sites reporting data. There was substantial variation in gestation at first review, although the mean attendance was at 24.8 ± 7.2 weeks. Among women with GDM, diet alone was used in 39.4% (28.8–57.3%) with significant variation in use of metformin alone (0.4–43.7%), metformin + insulin (1.5–23.4%) and insulin therapy alone (4.1–95.6%) (all *p* < 0.001). Elective and emergency caesarean delivery ranged from 3.6% to 23.7% and 3.5% to 21.2%, respectively (both *p* < 0.0001). Preterm birth (28 to <37 weeks) ranged from 3.7% to 9.4% (*p* < 0.05), large-for-gestational-age 10.3–26.7% (*p* < 0.0001), small-for-gestational-age 0–11.9% (*p* < 0.0001), admission to special care nursery 16.7–25.0% (*p* < 0.0001), neonatal hypoglycaemia (< 2.6 mmol/L) 6.0–27.0% (*p* < 0.0001), preeclampsia 2.0–5.2% (*p* < 0.0001), and shoulder dystocia 0–7.2% (*p* < 0.0001) all varied significantly across services. There was no significant difference in breastfeeding on discharge or jaundice requiring phototherapy. Major but not minor malformations varied across sites.

### 3.3. Pre-Gestational Diabetes

#### 3.3.1. Pre-Gestational Diabetes: Pregnancy Planning

Table 5 shows that for women with both T1D and T2D in pregnancy, recording measures of pregnancy planning were limited. Where data were available, recorded uptake varied significantly across services for women with pregestational diabetes including first trimester higher dose folate ranging from 28.6% to 100%, pregnancy planning ranging from 14.3% to 87.5% and gestation at first attendance from three to 33 weeks gestation. Only limited exposure (0.7–1.1%) was shown to statins and ACE inhibitors/angiotensin receptor blockers at conception. Oral anti-hyperglycaemic agents, inclusive of metformin, which was not reported separately, were in use at conception in 20.0–58.2% of women with T2D. A major concern is the higher mean HbA1c at conception of 7.7 ± 1.6% (61 ± 17 mmol/mol) and 7.3 ± 1.7% (56 ± 19 mmol/mol) among women with T1D and T2D, respectively. Between 59.6% and 100% of women with TID and 0–58.3% of women with T2D had a first trimester HbA1c > 6.5% (48 mmol/mol). However, these results exclude 10.6% of women with T1D and 46.2% of women with T2D without a documented early HbA1c. Complication screening ranged from 9.3% to 94.7% women having a first trimester urinary albumin:creatinine ratio. Prevalences of documented retinopathy and nephropathy in women with T1D ranged from 6.3% to 39.6% and 3.2–6.3%, respectively, and with T2D 0.7–7.9% and 5.3–20.0%, respectively. 

#### 3.3.2. Pre-Gestational Diabetes: Antenatal Management

The mean recorded gestational week at first visit to the participating diabetes service was beyond the recommended eight weeks of gestation [17] for women with both T1D and T2D (9.1 ± 5.3 and 11.1 ± 6.1 weeks respectively). The documentation of hypoglycaemia needing assistance was limited for all women with known diabetes (<5%). The HbA1c testing in the second trimester was limited at 26.2% (T1D) and 19% (T2D), although 89.9% and 59.4% respectively were completed in the third trimester. The HbA1c in the third trimester remained above target in >6.0% (42 mmol/mol) among most women with T1D (range 50.0–88.6%) and just under 50% of women with T2D (range 12.5–100%). Metformin use varied between 0 and 100% in women with T2D, while insulin pump use in women with T1D varied between 0 and 68% among reporting sites. Aspirin use for preeclampsia prophylaxis ranged between 0% and 59.0% across all women.

#### 3.3.3. Pre-Gestational Diabetes: Pregnancy Outcomes

Variable recording of birth outcomes occurred. While birth weight was available for 98.2% of infants, recording of several important neonatal outcomes was low. The reporting of malformations was limited, particularly among pregnancies complicated by T2D such that interpretation was difficult. Where recorded, rates of major pregnancy complications were high for women with both T1D and T2D. Elective caesarean rates ranged from 12.5–45.8% and emergency caesarean from 17.4% to 57.7% in T1D, and 0–73.7% and 0–48.3% in T2D. Gestational age at delivery was well recorded with marked variation in the prematurity rates (<37 weeks) of 7.5–52.1% in T1D and 13.6–31.6% in T2D (both *p* < 0.05), while birthweight ≥4000 g ranged from 14.3% to 37.5% and 0–23.6% respectively. LGA reporting was limited due to missing variables for the centile calculator (e.g., ethnicity). T1D data were relatively complete and showed significant variation in neonatal hypoglycaemia (12.5–87.5%) and admission to special care nursery (12.5–100%) (both *p* < 0.001).

## 4. Discussion

This pilot multi-centre audit involved the collection of de-identified data, testing of the internal governance process and benchmarking, and reporting of pregnancy care data from eleven volunteer diabetes health services across Australia and New Zealand. Our comparative analyses showed significant variation in diabetes care and pregnancy outcomes among services for women with GDM, T1D, and T2D. The reporting of pre-conception and antenatal care was limited for women with GDM and pre-existing diabetes, particularly for those with T2D. A large number of missing data items indicated variability between individual audits, lack of real time collection tools and absence of agreed indicators for measuring quality of care. 

For women with GDM, these 2020 results are roughly comparable to those from 2003 [14] including the gestation at birth (39 weeks), birthweight <2000 g (6% vs. 5%), elective caesarean section (12%) and shoulder dystocia (3%), with some possible reductions in birthweight ≥4000 g (9.6% vs. 14%), neonatal hypoglycaemia (16.9% vs. 20%), and emergency caesarean section (9.5% vs. 12%). However, the large variation in care and birth outcomes has continued. Since that first audit, the process for screening and diagnosing GDM has changed [19], new management approaches have been adopted in some services including metformin therapy, and a range of different models of care have been evolving [20]. It is possible that some sites missed women, particularly with diet-managed GDM, because they remained under general obstetric and/or private care and did not receive any local diabetes service support.

For women with pregestational diabetes, since the initial ADIPS benchmarking program in 2003 and a subsequent audit in 2004 across 10 Australian teaching hospitals, some aspects of care have probably improved [12,21], e.g., pre-pregnancy counselling and folate supplementation. However, while 31% (T1D) and 39% (T2D) had a caesarean delivery in the 2003 audit, and 63% had caesarean deliveries in the 2004 audit, >50% (T1D) and ~27% (T2D) had caesarean deliveries in the current audit. First trimester HbA1c may have improved in T2D but not T1D, with HbA1c 7.6% (60 mmol/mol) (T1D) and 7.8% (62 mmol/mol) (T2D) respectively in 2003, 7.1% (54 mmol/mol) overall in 2004 but 7.7% (61 mmol/mol) (T1D) and 7.3% (56 mmol/mol) (T2D) currently. Of additional concern is no apparent change in neonatal hypoglycaemia over this time with rates of 48% (T1D) and 30% (T2D) in 2003 compared to 53.3% (T1D) and 32.7% (T2D) now. Such limited changes also occurred in the national audit reported recently from the UK [11], which recommended health system changes to improve pre-pregnancy planning and antenatal care for women with pre-gestational diabetes [11,12]. One possible trend (depending on ascertainment) of interest is the increasing number of women with T2D: 49% in the first ADIPS audit, 55% in the 2004 audit, and currently accounting for 70% of women with pregestational diabetes.

The main challenge encountered during the implementation of this audit was the lengthy and complicated ethics approval process at some locations, which prevented several services from participating in the audit. Some services had to obtain state-based approval for accessing data and transferring them interstate. Some services had Aboriginal community-controlled organisations as partners and required approval from an indigenous reference group, which was not feasible within the timelines and governance processes of this audit. Obtaining such approval appropriately is obviously crucial in ensuring the trust of both health care providers and communities/patients in these areas. Coordinating the multiple layers of governance however made the audit process time consuming and complicated, but provided lessons for any future, sustainable widespread audit and benchmarking programme.

Although the audit tool provided useful information on antenatal care, problems were encountered when collating birth data. Endocrinologists/stand-alone diabetes services in close contact with women during their prenatal period held detailed pre-pregnancy care data but could not provide information following transfer of care to an antenatal service. Similarly, diabetes services are not necessarily integrated with antenatal care. 

Mismatches occurred between the variables listed in the audit tool and those that had already been collected by the participating services. Some had defined ethnicity based on the country-of-birth criteria and others reported only major ethnic groups, ignoring noticeable heterogeneity within each major group. For example, New Zealand but not Australian sites separated Māori and Pacific women while others did not separate East Asian and South East Asian women. This lack of consistency in ethnicity data coding made ethnicity classification difficult. Similarly, gestational hypertension and chronic hypertension were grouped into one category for some women when the service initially collected the data. For monitoring proteinuria, some services had measured urine protein to creatinine ratio more often than urine ACR especially in late pregnancy, but the PCR data were not included in the audit. Regarding data fields for the estimated date of confinement (EDC), the majority of sites reported initial EDC based on last menstrual period with a few sites basing EDC on dating US scan, however the data many have been collected by the antenatal clinic- indicating work needed in aligning communication/documentation between stand-alone diabetes and associated antenatal services. Significantly, data on hypoglycaemia needing assistance were missing for all services except one centre, and none of the services reported data for the number of hypoglycaemic episodes: it is unclear whether this was due to missing or data or that these events are now very rare. The high rates of congenital anomalies in these live births (with miscarriages and fetal loss excluded) suggest major malformation rates would be higher reinforcing the need for preconception care for women with known diabetes. A recent meta-analysis showed a 70% reduction in congenital malformation in women who received preconception care [22]. The large proportion of missing data on congenital malformation is another concern. Further, the current numbers may be incomplete because data on late-onset malformations are only available later in infancy. Some services offered aggregate data (i.e., summaries of the data they hold) and this was excluded in the audit but may need to be an option in the future. 

The current audit tool may require refinement in the future to separate some ethnic groups explicitly (e.g., Māori and Pacific) and metformin from other diabetes treatments at conception. Access to pre-pregnancy clinics [12] and the recent introduction of continuous glucose monitoring are also activities that need to be included to evaluate associations with outcomes and variation in use. However, one of the challenges of these audits is that data entry is time consuming, particularly if data is held in multiple locations and most centres rely on clinicians who already have heavy clinic loads to update the database. While it is desirable to collect as many important variables as possible, this has to be balanced against the feasibility at the various centres in terms of human resources. The data set will be reviewed before the next audit round to decide which need to be dropped, refined or added. Methods for data transfer (e.g., using services such as CloudStor) are also under review for sending de-identified data. Ideally, the data are entered into a system where the centre has ongoing access to reports and can share them between antenatal and diabetes services. Such a system will implement measures to harmonize data collection and validation processes among health services. Birth centre discharge summary forms could be updated to include such audit data which could be and sent to nominated diabetes care providers in addition to general practitioners.

The major strength of this study is that it is the largest national benchmarking audit of diabetes in pregnancy services across Australia and New Zealand. Diabetes services with a range of characteristics in major metropolitan cities in Australia and New Zealand as well as in rural and private care settings were included. A major limitation of the audit is the lack of data on pregnancies ending before 28 weeks gestation including miscarriage, stillbirth or induced abortion and the audit will underestimate the proportion of pregnancies with some adverse outcomes (e.g., malformations, pre-eclampsia) as a result. These data should be collected comprehensively in future audits. Another limitation was a difficulty defining a denominator in some analyses due to missing/uncoded data. There was some variation in GDM diagnosis: the study population included women with GDM diagnosed based on ADIPS 2014 [23], ADIPS 1998 [24], and New Zealand GDM criteria [25]. Further limitations were that these were voluntary sites and they may not be representative of services across Australia and New Zealand (as evidenced by relatively low proportion of women of European descent compared with national data). Similarly, as in 2003 and 2004, the audits were from a limited number of sites with modest numbers of patients with different sites in each audit. Nonetheless the findings do illustrate the usefulness of periodic voluntary auditing as it gives an indication of changes over time. The considerable variation between sites in some outcomes may be caused partly by the differences in population characteristics that they serve, and no attempt has been made to assess for population differences as we did not want to potentially identify sites by presenting data on ethnic background or diagnostic blood glucose data. Further, individual outcomes will be influenced by the completeness of the data submitted for analysis. Missing data may reflect the fact that a particular field or fields is/are not recorded in the database, and not necessarily that they are not part of patient care. 

## 5. Conclusions

Our national pilot audit provides information on how quality indicators in perinatal diabetes care have been followed and documented by diabetes services for women with pre-existing diabetes and GDM. Although the benchmarking report across sites is not shown in this paper, our findings indicate an under-recording of the diabetes care provided to these women and evidence that improvement in care is needed even among these volunteer sites with a major interest in optimising pregnancy outcomes. There is a particular need for targeted and improved pregnancy preparation for women with T2D. As quality perinatal care is integral for the survival and well-being of the fetus, an ongoing national audit is necessary to improve pregnancy care for women affected by hyperglycaemia according to the standards set by the ADIPS. This preliminary, and essentially exploratory audit, highlights that a national clinical audit with defined and regulated outcomes, is feasible and urgently needed with communication between both maternity and diabetes services.

## Figures and Tables

**Table 1:** ADIPS key recommendations that were audited for women with diabetes [17].

No	Best Practice Recommendations for Women with Diabetes
1	All pregnancies should be intended pregnancies.
2	Pre-pregnancy counselling regarding risks should be provided.
3	Teratogenic medications should be avoided before conception and during pregnancy.
4	Blood glucose should be near normal before conception and during pregnancy.
5	Women should receive high dose (2.5–5 mg daily) folate supplementation starting 3 months prior to conception until 12 weeks gestation.
6	Oral hypoglycaemic agents should be reviewed prior to conception.
7	First antenatal visit should occur early in pregnancy, before 8 weeks of gestation.
8	Glycaemic control should be balanced against the risk of hypoglycaemia in early pregnancy.
9	Women with diabetes should be monitored for signs or progression of diabetes microvascular complications.
10	Vaginal birth should be encouraged unless there are obstetric or fetal indications for caesarean section.
11	Breastfeeding should be encouraged for all women with diabetes.

ADIPS = Australasian Diabetes in Pregnancy Society.

**Table 2:** Final audit tool used.

Category	Data Items
For all women	Patient ID, date of birth, date of referral, EDC, estimated EDC by ultrasound, ethnic group, gestation at first review, gravidity, parity, height, pre-pregnancy weight, booking weight, smoking status, chronic hypertension, gestational hypertension, preeclampsia, delivery method, baby’s date of birth, gestational age at delivery, baby sex, birth weight, APGAR at 5 mins, shoulder dystocia, other birth trauma, admission to SCN/NICU, neonatal hypoglycaemia <2.6 mmol/L, jaundice needing phototherapy, minor congenital malformation, major congenital malformation, breast feeding on discharge.
Women with GDM	OGTT fasting, OGTT 1 h, OGTT 2 h, HbA1c at GDM diagnosis, GDM treatment.
Women with type 1 or type 2 diabetes	Planned pregnancy, OHA at conception, statin at conception, ACE/ARB at conception, folate (1st trimester folate dose), aspirin use, retinopathy, nephropathy, hypoglycaemia needing assistance, number of hypoglycaemic episodes needing assistance, eGFR, urine ACR, HbA1c % trimester 1, HbA1c % trimester 2, HbA1c % trimester 3, type 1 diabetes treatment, type 2 diabetes treatment.

EDC = Estimated date of confinement; SCN = Special care nursery; NICU = Neonatal intensive care unit; OHA = Oral hypoglycaemic agents; ACE = Angiotensin-converting enzyme inhibitors; ARB = Angiotensin II receptor blockers; eGFR= estimated glomerular filtration rate, ACR = Albumin creatinine ratio, GDM = Gestational diabetes mellitus; OGTT = Oral glucose tolerance test.

**Table 3:** Baseline characteristics of the study population (live singleton births only).

Characteristics	N	Mean ± SD or n (%) (Range) @	Range (N, Mean or % Across All Sites)	Number of Sites Missing 100% Data (Sites Not Reporting/Collecting) ^†^	Missing Values	
					N	% Missing Range Across All Sites
Number of women	10,144	--	59–2426	0	--	--
GDM	8696	--	54–2397	0	--	--
Type 1 diabetes	435	--	7–295	0	--	--
Type 2 diabetes	1013	--	19–428	0	--	--
Age (years)	10,114	32.3 ± 5.5 (15–51)	31.0–35.1	0	30	0–11.9
Gravidity	10,065	2.9 ± 1.9 (1–22)	2.4–3.3	0	79	0–8.9
Parity	10,066	1.3 ± 1.4 (0–11)	0.8–1.6	0	78	0–16.9
Pre-pregnancy weight (kg)	6471	74.7 ± 21.1 (34.0–185.0)	68.5–89.8	3	3673	0–100
Mean Pre-pregnancy BMI (kg/m^2^)						
GDM	6060	28.1 ± 7.0 (26.2–32.7)	26.2–32.7	5	2636	30.3
Type 1 diabetes	215	26.0 ± 5.4 (22.8–30.7)	22.8–30.7	5	320	73.6
Type 2 diabetes	244	31.5 ± 7.1 (29.9–38.6)	29.1–38.6	4	769	75.9
Booking weight (kg)	6191	84.0 ± 23.5 (33.9–193.0)	71.3–88.6	4	3953	0–99.9
History of hypertension	296	6.7	1.9–12.9	2	5739	0–100
Smoking	566	9.1	2.3–15.1	2	3919	0–100

@ percentage of all possible values. ^†^ Sites missing 100% of data were removed from the denominator; For categorical data, if the sites had reported only “Yes” values and the remaining cells were blank/missing, these were interpreted as “No”. BMI = Body mass index; GDM = Gestational Diabetes Mellitus.

**Table 4:** Pregnancy care and obstetric outcomes of women with gestational diabetes mellitus.

Variables	Mean ± SD or N/Total Available Data (%)@	Range Across Sites	Number of Sites Missing 100% Data	Missing Across Sites Reporting N/(%) ^†^
Gestation at first review (weeks)	24.8 ± 7.2	22.1–26.7 ***	4	6195 (71.2)
Diet Only	2082/5278 (39.4)	28.8–57.3 **	1	3418 (39.3)
Metformin use only	991/5278 (18.8)	0.4–43.7 **	1	3418 (39.3)
Insulin use only	1671/5278 (31.7)	4.1–95.6 **	1	3418 (39.3)
Cesarean delivery: (a) Elective (b) Emergency (c) Any	1030 (12.1) 707 (9.5) 1771 (20.3)	3.6–23.7 *** 3.5–21.2 *** 12.3–42.6 ***	2 2 0	195 (2.2) 1225 (14.1) 0
Gestational age at delivery (weeks)	38.6 ± 1.5	38.2–38.9 ***	0	174 (2.0)
Late preterm (34–37 weeks)	651/8696 (7.5)	3.7–9.4 *	0	174 (2.0)
Very preterm/moderately preterm (<34 weeks)	128/8696 (1.5)	0.2–2.2 *	0	174 (2.0)
Birth weight ≥4.0 kg ≤2.5 kg	819/8526 (9.6) 509/8526 (6.0)	5.9–13.9 *** 1.9–6.8 #	0 0	170 (2.0) 170 (2.0)
LGA	754/5759 (13.1)	10.3–26.7 ***	2	2937 (33.8)
SGA	538/5759 (9.3)	0–11.9 ***	2	2937 (33.8)
Preeclampsia	110/3590 (3.1)	2.0–5.2 ***	2	5106 (58.7)
Shoulder dystocia	92/3321 (2.8)	0.0–7.2 ***	4	4629 (53.2)
Admission to SCN/NICU	710/3347 (21.2)	16.7–25.0 ***	3	5378 (61.8)
Neonatal hypoglycaemia <2.6 mmol/L	690/4079 (16.9)	6.0–27.0 ***	3	4599 (52.9)
Jaundice needing phototherapy	122/3261 (4.9)	4.7–5.3 #	5	5435 (62.5)
Minor congenital malformations Major congenital malformations	22/3321 (0.7) 61/3321 (1.8)	0.0–1.2 # 0.9–3.4 **	4 4	4616 (53.1) 4629 (53.2)
Breast feeding on discharge	795/1273(62.5)	57.4–62.7 #	6	7279 (83.7)

@ Percentage of all possible values; † Sites missing 100% of data were removed from denominator; For categorical data, if the sites had reported only “Yes” values and the remaining cells were blank/missing, these were interpreted as “No”. SCN = Special care nursery; NICU = Neonatal intensive care unit; LGA = Large for Gestational Age; SGA = Small for Gestational Age; *p* values refer to comparisons between sites; # Nonsignificant; * *p* < 0.05; ** *p* < 0.001; *** *p* < 0.0001.

**Table 5:** Pregnancy care and obstetric outcomes of women with pre-existing diabetes.

Variables	Type 1 Diabetes N = 435				Type 2 Diabetes N = 1013			
	N/(%) Mean ± SD or %@	Range Across Sites	Number of Sites Missing 100% Data	Missing Values Across Sites Reporting N/(%) ^†^	N/(%) Mean ± SD or %@	Range	Number of Sites Missing 100% Data	Missing Values Across Sites Reporting N/(%) ^†^
Gestation at first review (weeks)	9.1 ± 5.3	3–27 ***	4	343 (78.9)	11.1 ± 6.1	3.5–33 *	5	862 (85.1)
Pregnancy planning	65/101 (64.4)	14.3–87.5 ***	3	339 (77.9)	97/207 (46.9)	20.0–63.2 *	4	812 (80.2)
OHA/GLP1RA on conception	1/94 (1.1)	--	4	342 (78.6)	100/210 (47.6)	20.0–58.2 **	5	824 (81.3)
Statin at conception	0/93 (0.0)	--	4	342 (78.6)	1/152 (0.7)	--	5	862 (85.1)
ACEI/ARB at conception	1/93 (1.1)	--	4	342 (78.6)	1/152 (0.7)	--	5	863 (85.2)
First trimester Folate (5mg)	92/101 (91.1)	28.6–100 ***	3	335 (77.0)	178/207 (86.0)	57.9–100 ***	4	810 (80.0)
Retinopathy	77/428 (18.0)	6.3–39.6 **	1	49 (11.3)	16/673 (2.4)	0.7–7.9 *	2	439 (43.3)
Nephropathy	21/428 (4.9)	3.2–6.3 #	1	40 (9.2)	95/991 (9.6)	5.3–20.0 ***	1	97 (9.6)
Urine ACR	145 (33.3)	26.4–86.8 #		290 (66.7)	196 (19.3) #	9.3–94.7		817 (80.7)
Aspirin use in pregnancy	215/428 (50.2)	(13.2–59.0) ***	1	343 (78.9)	178/673 (26.4)	(0–55.6) ***	2	862 (85.1)
Hypoglycaemia needing assistance	0/45 (0.0)	--	6	390 (89.7)	1/24 (4.2)	--	7	989 (97.6)
HbA1c 1st trimester	7.7 ± 1.6	5–16.5 **	2	46 (10.6)	7.3 ± 1.7	4.2–14.0 *	3	468 (46.2)
HbA1c 2nd trimester	6.6 ± 1.1	4.5–11.1 **	1	321 (73.8)	6.1 ± 1.1	4.3–10.4 *	2	821 (81.0)
HbA1c 3rd trimester	6.8 ± 1.0	4.8–11.0 *	1	44 (10.1)	6.3 ± 1.1	4.0–12.6 **	2	411 (40.6)
HbA1c 1st trimester >6.5% (48mmol/mol)	78.4	59.6–100 *	2	46 (10.6)	54.6	0–58.3 **	3	469 (46.3)
HbA1c 2nd trimester >6.0% (42 mmol/mol)	69.9	50.0–82.4 #	1	322 (74.0)	41.7	5.9–62.5 #	2	821 (81.0)
HbA1c 3rd trimester >6.0% (42 mmol/mol)	77.2	50.0–88.6 #	1	45 (10.3)	48.5	12.5–100 *	2	411 (40.6
Diet Only	--	--	--	--	3/568 (0.5)	0–0.5	1	445 (43.9)
Metformin use only	--	--	--	--	158/568 (27.8)	0–100	1	445 (43.9)
Insulin use only	--	--	--	--	132/568 (23.2)	0–100	1	445 (43.9)
Metformin and Insulin use	--	--	--	--	275/568 (48.4)	0–79.9	1	445 (43.9)
Insulin pump	49/134 (36.6)	0–68.8	1	301 (69.2)	--	--	-	--
Cesarean delivery: (a) Elective (b) Emergency	50/137 (36.5) 36/87 (41.4)	12.5–45.8 # 17.4–57.7 **	1 1	298 (68.5) 348 (80.0)	97/574 (16.9) 49/477 (10.3)	0–73.7 *** 0–48.3 ***	1 1	439 (43.3) 536 (52.9)
Gestational age at delivery (weeks)	36.4 ± 1.7	28.1–40.2*	0	1 (0.2)	37.3 ± 1.8	27.3–41.3 ***	0	12 (1.2)
Late preterm (34–37 weeks)	208/435 (47.9)	7.5–52.1 *	0	1 (0.2)	227/1013 (22.4)	13.6–31.6 *	0	12 (1.2)
Very preterm/moderately preterm ( < 34 weeks)	36/435 (8.3)	0.5–28.6 #	0	1 (0.2)	58/1013 (5.7)	4.5–7.3 #	0	12 (1.2)
Birth weight ≥ 4.0 kg ≤ 2.5 kg	103/432 (23.8) 37/432 (8.6)	14.3–37.5 # 2.1–14.3 #	0 0	3 (0.7) 3 (0.7)	157/1003 (15.7) 101/1003 (10.1)	0–23.6 ** 5.3–15.8 #	0 0	10 (1.0) 10 (1.0)
LGA	70/112 (62.5)	20.0–85.7 #	1	323 (74.3)	58/225 (25.8)	15.8–33.3 #	2	788 (77.8)
SGA	5/112 (4.5)	0–16.7 #	1	323 (74.3)	29/225 (12.9)	0–20.0 #	2	788 (77.8)
Preeclampsia	76/425 (17.9)	14.9–28.6#	0	10 (2.3)	74/681 (10.9)	5.0–33.3*	1	332 (32.8)
Shoulder dystocia	2/102 (2.0)	0–3.1 #	2	336 (77.2)	1/158 (0.6)	-	3	860 (84.9)
Admission to SCN/NICU	279/428 (65.2)	12.5–100 ***	1	14 (3.2)	273/676 (40.4)	21.8–50.0 *	2	362 (35.7)
Neonatal hypoglycaemia <2.6 mmol/L	232/435 (53.3)	12.5–87.5 ***	0	24 (5.5)	227/695 (32.7)	10.5–52.6 **	1	390 (38.5)
Jaundice needing phototherapy	44/140 (31.4)	12.5–51.3 **	1	301 (69.2)	54/267 (20.2)	5.3–40.0 #	2	770 (76.0)
Minor congenital malformations Major congenital malformations	5/94 (5.3) 3/94 (3.2)	6.0–28.6 * 2.1–16.7 #	3 3	344 (79.1) 343 (78.9)	9/103 (8.7) 2/103 (1.9)	0–15.8 # 0–5.3 #	4 4	918 (90.6) 918 (90.6)
Breast feeding on discharge	98/125(78.4)	57.1–93.8 *	3	313 (72.0)	141/190 (74.2)	20.0–89.5 *	4	831 (82.0)

@ Percentage of all possible values; † Sites missing 100% of data were removed from the denominator; For categorical data, if the sites had reported only “Yes” values and the remaining cells were blank/missing, these were interpreted as “No”. OHA = Oral hypoglycaemic agents including metformin; ACEI= Angiotensin converting enzyme inhibitors; ARB = Angiotensin receptor blockers; ACR = Albumin creatinine ratio; SCN = Special care nursery; NICU = Neonatal intensive care unit; LGA = Large for Gestational Age; SGA = Small for Gestational Age. *p* values refer to comparisons between sites within type of diabetes; # Nonsignificant; * *p* < 0.05; ** *p* < 0.001; *** *p* < 0.0001.

## Data Availability

The data are not publicly available due to privacy and ethical restrictions.

## References

[1] Yuen L., Saeedi P., Riaz M., Kururanga S., Divakar H., Levitt N., Xilin Y., Simmons D. (2019). IDF Diabetes Atlas: Projections of the prevalence of hyperglycaemia in pregnancy in 2019 and beyond: Results from the International Diabetes Federation Diabetes Atlas, 9th edition. Diabetes Res. Clin. Pract..

[2] The HAPO Study Cooperative Research Group (2008). Hyperglycemia and Adverse Pregnancy Outcomes. N. Engl. J. Med..

[3] Diabetes Control and Complications Trial Research Group (1996). Pregnancy outcomes in the diabetes control and complications trial. Am. J. Obstet. Gynecol..

[4] Australian Institute of Health and Welfare (2019). Diabetes in Pregnancy 2014–2015. Bulletin no. 146. Cat. no. CDK 7.

[5] Australian Institute of Health and Welfare (2010). Diabetes in Pregnancy: Its Impact on Australian Women and Their Babies. Diabetes Series no. 14. Cat. no. CVD 52.

[6] Kirke A.B., Atkinson D., Moore S., Sterry K., Singleton S., Roxburgh C., Parrish K., Porter C., Marley J.V. (2019). Diabetes screening in pregnancy failing women in rural Western Australia: An audit of oral glucose tolerance test completion rates. Aust. J. Rural. Health.

[7] Mackin S.T., Nelson S.M., Kerssens J.J., Wood R., Wild S., Colhoun H.M., Leese G.P., Philip S., Lindsay R.S. (2018). SDRN Epidemiology Group. Diabetes and pregnancy: National trends over a 15 year period. Diabetologia.

[8] Australian Atlas of Healthcare Variation 2017: 3.0 Women’s Health and Maternity—Introduction and Key Recommendations. https://www.safetyandquality.gov.au/sites/default/files/migrated/3.0-Introduction-and-key-recommendations.pdf.

[9] Simmons D., Oats J., Hod M. (2008). Diabetes in Pregnancy in New Zealand. Textbook of Diabetes and Pregnancy.

[10] Murphy H.R., Bell R., Cartwright C., Curnow P., Maresh M., Morgan M., Sylvester C., Young B. (2017). Improved pregnancy outcomes in women with type 1 and type 2 diabetes but substantial clinic-to-clinic variations: A prospective nationwide study. Diabetologia.

[11] Murphy H.R., Howgate C., O’Keefe J., Myers J., Morgan M., Coleman M.A., Jolly M., Valabhji J., Scott E.M., Knighton P. (2021). National Pregnancy in Diabetes (NPID) advisory group. Characteristics and outcomes of pregnant women with type 1 or type 2 diabetes: A 5-year national population-based cohort study. Lancet Diabetes Endocrinol..

[12] Simmons D. (2021). Adverse pregnancy outcomes in women with type 1 or type 2 diabetes. Lancet Diabetes Endocrinol..

[13] Lee A.S., Colagiuri S., Flack J.R. (2018). Successful implementation of diabetes audits in Australia: The Australian National Diabetes Information Audit and Benchmarking (ANDIAB) initiative. Diabet. Med..

[14] Simmons D., Cheung N.W., McIntyre D., Flack J.R., Lagstrom J., Bond D., Johnson E., Wolmarans L., Wein P., Sinha A.K. (2007). The ADIPS Pilot National Diabetes in Pregnancy Audit Project. Aust. N. Z. J. Obstet. Gynae..

[15] Feig D.S., Corcoy R., Jensen D.M., Kautzky-Willer A., Nolan C.J., Oats J.J.N., Sacks D.A., Caimari F., McIntyre H.D. (2015). Diabetes in pregnancy outcomes: A systematic review and proposed codification of definitions. Diabetes Metab. Res. Rev..

[16] Sinha I.P., Smyth R.L., Williamson P.R. (2011). Using the Delphi technique to determine which outcomes to measure in clinical trials: Recommendations for the future based on a systematic review of existing studies. PLoS Med..

[17] Rudland V.L., Price S.A.L., Hughes R., Barrett H.L., Lagstrom J., Porter C., Britten F.L., Glastras S., Fulcher I., Wein P. (2020). ADIPS 2020 guideline for pre-existing diabetes and pregnancy. Aust. N. Z. J. Obstet. Gynaecol..

[18] Australian Government Australian Institute of Health and Welfare. Metadata Online Registry. https://meteor.aihw.gov.au/content/index.phtml/itemId/181414-title=About.

[19] Simmons D. (2020). The benefits of the use of the new International Association of Diabetes in Pregnancy Study Groups guidelines for gestational diabetes mellitus. Aust. N. Z. J. Obstet. Gynaecol..

[20] Sina M., Cade T.J., Flack J., Nolan C.J., Rajagopal R., Wong V., Burcher L., Barry A., Gianatti E., McCarthy A. (2020). Antenatal models of care for women with gestational diabetes mellitus: Vignettes from an international meeting. Aust. N. Z. J. Obstet. Gynaecol..

[21] McElduff A., Ross G.P., Lagström J.A., Champion B., Flack J.R., Lau S.M., Moses R.G., Seneratne S., McLean M., Cheung N.W. (2005). Pregestational Diabetes and Pregnancy: An Australian experience. Diabetes Care.

[22] Wahabi H.A., Fayed A., Esmaeil SElmorshedy H., Titi M.A., Amer Y.S., Alzeidan R.A., Alodhayani A.A., Saeed E., Bahkali K.H., Kahili-Heede M.K. (2020). Systematic review and meta-analysis of the effectiveness of pre-pregnancy care for women with diabetes for improving maternal and perinatal outcomes. PLoS ONE.

[23] Nankervis A., McIntyre H.D., Moses R., Ross G.P., Callaway L., Porter C., Jeffries W., Boorman C., De Vries B., McElduff A. ADIPS Consensus Guidelines for the Testing and Diagnosis of Gestational Diabetes Mellitus in Australia. http://www.adips.org/downloads/ADIPSConsensusGuidelinesGDM-03.05.13VersionACCEPTEDFINAL.pdf.

[24] Hoffman L., Nolan C., Wilson J.D., Oats J.J., Simmons D. (1998). The Australasian Diabetes in Pregnancy Society. Gestational diabetes mellitus—Management guidelines. Med. J. Aust..

[25] Ministry of Health (2014). Screening, Diagnosis and Management of Gestational Diabetes in New Zealand: A Clinical Practice Guideline.

